# Revealing homogeneous plastic deformation in dendrite-reinforced Ti-based metallic glass composites under tension

**DOI:** 10.1038/srep42598

**Published:** 2017-02-14

**Authors:** F. F. Wu, J. S. Wei, K. C. Chan, S. H. Chen, R. D. Zhao, G. A. Zhang, X. F. Wu

**Affiliations:** 1 School of Materials Science and Engineering, Liaoning University of Technology, Jinzhou 121001, China; 2 Advanced Manufacturing Technology Research Centre, Department of Industrial and Systems Engineering, Hong Kong Polytechnic University, Kowloon, Hong Kong, China

## Abstract

The tensile plastic deformation of dendrite-reinforced Ti-based metallic glass composites (MGCs) was investigated. It was found that there is a critical normalized strain-hardening rate (NSHR) that determines the plastic stability of MGCs: if the NSHR is larger than the critical value, the plastic deformation of the MGCs will be stable, i.e. the necking and strain localization can be effectively suppressed, resulting in homogeneous plastic elongation. In addition, dendrite-reinforce MGCs are verified as being intrinsically ductile, and can be used as good coatings for improving the surface properties of pure titanium or titanium alloys. These findings are helpful in designing, producing, and using MGCs with improved performance properties.

Bulk metallic glasses (BMGs) are regarded as potential candidates for structural components due to their high strength and other unique physical and chemical properties^1^,^2^,^3^. However, most BMGs have been found to have very limited tensile ductility and fracture toughness, which results from the fast and unstable propagation of shear bands, which severely limits their application in engineering fields^4^,^5^,^6^,^7^. It is noted that, at the micron scale, the shear strain in a shear band of a metallic glass (MG) is larger than 10^2^, indicating MGs should be intrinsically ductile at the micron scale^8^. This has been verified by reports on the plastic deformation of MGs with sample sizes decreasing to the micrometer or nanometer scale^5^,^9^,^10^,^11^,^12^.

Over the past decade, it is found that introducing an *in-situ* ductile or soft phase of the body-centered cubic (*bcc) β* dendrite can effectively tailor the glassy matrix into number of patches at the micron size, and effectively inhibits the fast and unstable propagation of a major shear band. This then activates multiple tiny shear bands in the glassy matrix, which significantly improves the tensile ductility and fracture toughness of MG composites (MGCs)^13^,^14^,^15^. However, when they are subjected to tensile loading, MGCs always exhibit an inhomogeneous plastic deformation mode with strong strain localization or necking after yielding^16^,^17^. The strong necking phenomenon severely decreases the safety of MGCs when used in structural fields, which inhibits their use as structural materials. Therefore, how to stabilize the plastic deformation of *β* dendrite reinforced Ti-based MGCs is important and urgent for their engineering applications. However, the strong necking of *β* dendrite reinforced Zr- or Ti-based MGCs is not well understood.

In this work, a series of Ti-based MGCs (*β*-MGCs) with different *β* dendrite volume fraction were prepared. By investigating their mechanical responses under tension, the deformation mechanism was revealed as the key to understanding the plastic stability of *β* dendrite reinforced Ti-based MGCs.

## Results

### Microstructure and tensile plastic deformation of β-MGCs

**Figure 1:**
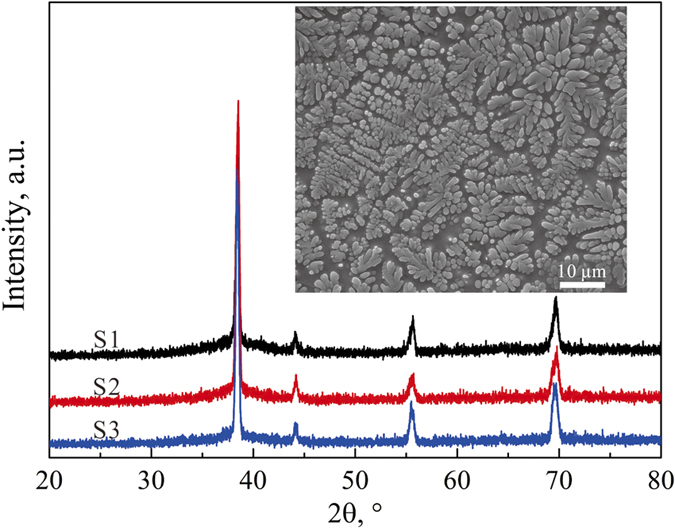
XRD patterns showing the mixture of amorphous and body-centered cubic crystalline structure of the metallic glass composites S1, S2, and S3. The inset is a typical SEM image indicating the microstructure of the metallic glass composites S1. The dendrites are the body-centered crystalline, and the matrix is amorphous.

**Figure 2:**
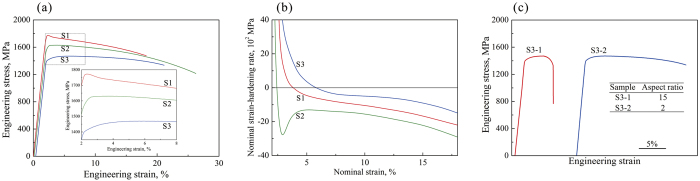
(**a**) Engineering stress-strain curves of the MGCs subjected to tension. (**b**) Corresponding nominal strain-hardening rate of the MGCs. (**c**) Engineering stress-strain curves of samples S3 with aspect ratio of 2 and 15, respectively.

**Figure 3:**
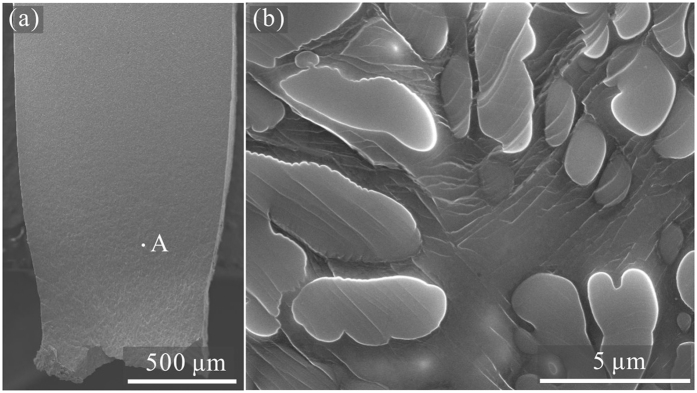
(**a**) Typical SEM image showing the necking phenomenon in the S1 subjected to tensile deformation. (**b**) Deformation feature close the necking region of the S1.

The XRD patterns of the *β-*MGCs had a mixture of *bcc* and amorphous phases, as shown in Fig. 1. The SEM images indicate that the secondary dendrite arm is about 3 μm in size on average (see the inset of Fig. 1). Based on the OM and SEM images, the volume fractions of the *β* dendrites were measured to be about 56 ± 6%, 70 ± 3%, and 81 ± 5% for the samples S1, S2, and S3, respectively. Figure 2 shows the typical engineering stress-strain curves of *β*-MGCs. Sample S1 yielded at 1636 MPa, followed by limited strain hardening to 1772 MPa at a plastic strain of 0.3%. The stress then decreased continuously till final fracture, which means the sample S1 underwent unstable plastic deformation after yielding. The SEM image demonstrates that sample S1 suffered from a strong necking or strain localization before the final fracture, as shown in Fig. 3(a). The area reduction in the thinnest neck region was 33.2%, and the derived corresponding true strain was 0.497. Clear large plastic deformation occurred in the *β* dendrites and the MG matrix. Sample S2 yielded at a stress of 1497 MPa, and then slightly strain-hardened to 1630 MPa at a plastic strain of 1.93%. Similar to sample S1, sample S2 also underwent unstable plastic deformation after yielding. The area reduction in the thinnest neck region of sample S2 was 45.7% on average, and the calculated true strain was 0.842. Sample S3 yielded at a stress of 1368 MPa, and then strain-hardened to 1470 MPa at a plastic strain of 3.46%. The stress decreased continuously till final fracture, showing that sample S3 also experienced unstable plastic deformation after remarkable strain-hardening. The area reduction in the thinnest neck region of S3 was 27.9%, and the corresponding true strain was 0.387.

It is worth noting that the present work was limited by the sample dimensions, especially the aspect ratio. A complete plastic deformation and fracture under necking can be achieved only if the aspect ratio of the sample is large enough and the sample’s elongation is larger than the length of the neck region^18^. If this critical condition is not met, the necking is confined and an extended plastic strain will be induced. To clarify the aspect ratio effect, samples of S3 with larger aspect ratio of 15 were tested under tension (due to sample preparation limitation, larger aspect ratio, such as 40 can’t be realized currently). It was found that the necking deformation was strongly dependent on the aspect ratio, as shown in Fig. 2c. Apparently, the plastic deformation after necking is remarkably decreased by the increasing aspect ratio.

### Tensile plastic deformation of bimetals composed of β-MGC and pure titanium

**Figure 4:**
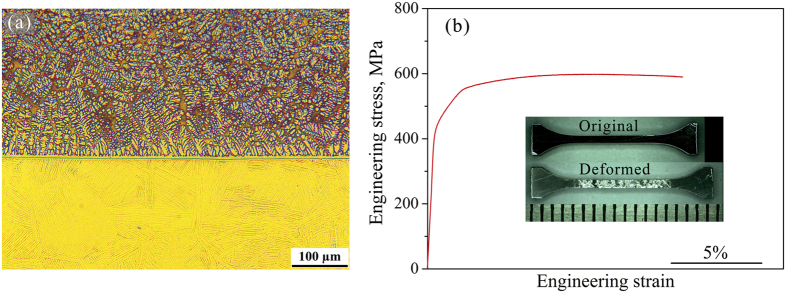
(**a**) Microstructure of the bimetal composed of the *bcc* dendrite reinforced Ti-based MGC and pure titanium (PT). (**b**) Engineering stress-strain curve of the MGC/PT bimetal. The inset shows the MGC/PT bimetal before and after tensile deformation.

Figure 4a shows the microstructure of a bimetal composed of *β*-MGC and pure titanium: the top layer is a *β* dendrite reinforced Ti-based MGC with composition Ti_45.4_Zr_26.4_Mo_6_Cu_9.4_Be_12.8_ (S1), and the lower substrate is pure titanium (PT). The interfacial layer between the MGC and the PT consists of a *β* Ti alloy with a bcc structure. The secondary dendrite arm in the *β*-MGC layer is about 5 um on average. The volume fraction of the *β* dendrites in the *β*-MGC layer is about 60%, measured from the OM and SEM images. The grain size of the PT substrate is about 150 ± 20 μm on average. Combined with the PT substrate, the *β-*MGC bimetal showed quite different plastic deformation behavior, as shown in Fig. 4b. The bimetals firstly yielded at 420 ± 15 MPa due to the plastic deformation of the PT, and secondly yielded at 660 ± 15 MPa due to the plastic deformation of the MGC. The bimetals then underwent remarkable homogeneous plastic deformation before the final fracture. After undergoing clear strain-hardening, the samples reached an ultimate strength of 732 ± 20 MPa. It was demonstrated that the *β-*MGC confined by PT could homogeneously deform, approaching a plastic strain of more than 13%. The necking of the monolithic MGC was effectively controlled and minimized by the PT, and no necking could be found in the fracture region, as shown in the inset of Fig. 4b.

## Discussion

### Normalized strain-hardening rate

**Figure 5:**
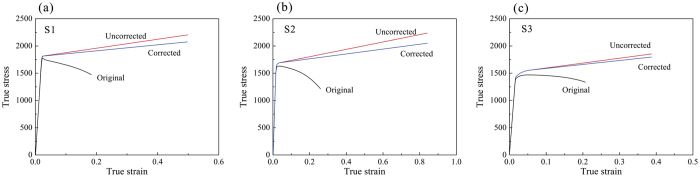
True stress-strain curves of (**a**) S1, (**b**) S2, and (**c**) S3 calculated from the corresponding load-displacement curves and the measured dimension of the necking.

According to the nominal stress-strain curves in Fig. 2a, it seems that all the MGC samples S1, S2, and S3 underwent obvious strain softening. However, by calculating the actual area and the load, the nominal true stresses *S*_*av*_ at the thinnest neck region were calculated to be 2207 MPa, 2236 MPa, and 1856 MPa for the samples S1, S2, and S3, respectively. These values are significantly higher than their corresponding ultimate tensile stresses of 1772 MPa, 1630 MPa, and 1470 MPa, indicating a remarkable strain hardening phenomenon, as shown in Fig. 5. However, due to the strong triaxial stress state in the neck region, the true stress *S* should be corrected by the Bridgeman formula^19^


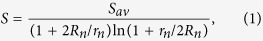


where *R*_*n*_ is the radius of curvature of the neck, and *r*_*n*_ is the radius of the cross-section in the thinnest part of the neck region. The value of *r*_*n*_ can be approximated to half the average length of the section for a rectangular specimen. By substituting the measured values into Eq. 1, the corrected true stress *S* of sample S2 was 2049 MPa, which is 8.3% smaller than the average stress *S*_*av*_. The true stress-strain curve of sample S2 is plotted as Fig. 5(b). The original curve is directly calculated from the engineering stress-strain curve, which is incorrect and invalid once necking begins. The uncorrected curve is plotted by recalculating the stress at the thinnest necking area, which is slightly larger than the corrected one due to the triaxial stress state. From the corrected value, it is shown that an obvious weak strain-hardening rate exists in sample S2. The average strain-hardening rate is about 440 MPa, which is relatively small in contrast with the yield strength (1490 MPa). Similarly, the average strain-hardening rates for samples S1 and S3 are calculated to be 553 MPa and 729 MPa, respectively. It is clear that the all the three MGCs underwent strain-hardening but not strain-softening during the necking process. For sample S1, due to there being no homogeneous plastic deformation, it is difficult to calcaluate the intial strain hardening rate. However, samples S2 and S3 have obvious homogeneous plastic deformation, therefore the initial strain-hardening rates before necking can be derived as 1690 MPa and 1552 MPa on average, respectively, which is much larger than that during the necking process.

The present and reported results^13^,^14^,^15^,^20^ show that the *β-*MGCs always demonstrate strong unstable plastic deformation after yielding, resulting in necking behavior. Even though the volume fraction of the *β* dendrites is as high as 70%^15^,^21^,^22^, there is still no homogeneous plastic deformation in *β*-MGCs. Usually, the macroscopic homogeneous plastic deformation is composed of numerous micro inhomogeneous deformations, *i.e.* the plastic deformation does not occur simultaneously in the whole gauge region^19^. The micro inhomogeneous deformation can be named after micro plastic unit here. In the case of homogeneous plastic deformation, the localized necking in the plastic unit can be inhibited by its strain-hardening. Therefore, the plastic deformation can be transferred to the neighboring undeformed plastic units. These processes are reproduced and homogeneous plastic deformation can be realized. However, if the necking in the plastic unit can not be counterbalanced by its strain-hardening, it will become thinner and form macroscopic necking, as shown in the final stage of most ductile metals and alloys^19^, as well as *β*-MGCs^13^,^14^,^15^,^20^,^21^,^22^. Therefore, in the following, the necking condition in the micro plastic unit for *β*-MGCs is discussed. Firstly, it is hypothesized that the micro plastic unit has dimensions of *L*_0_* × D*_0_ (length × diameter). Though a specimen is deformed under continuous loading or displacement, the deformation in metallic glasses always responds by an abrupt burst, which is related to the shear events such as the formation of a shear transformation zone and the initial shear banding^23^. It is presumed that this deformation burst is homogeneously distributed in the plastic unit, and the deformation burst lengthens the plastic unit with an increment of Δ*L*. Therefore, the true strain of the individual micro plastic strain unit can be expressed as^19^,^24^


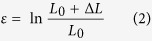


Correspondingly, the diameter of the plastic unit is reduced to *D*′, and the length of the deformed plastic unit is elongated to *L*′ = *L*_0_ + Δ*L*. In this case, the active strength of the deformed plastic unit is *σ*′ = *σ*_0_ + Δ*σ*, where *σ*_0_ and Δ*σ* are the original yield strength before plastic deformation and the strain-hardening induced strength increment, respectively. Then the strain-hardening rate *θ* is obtained as^24^:


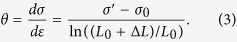


Dividing Eq. 3 by *σ*_0_, we get the initial normalized strain-hardening rate^24^:


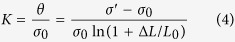


**Figure 6:**
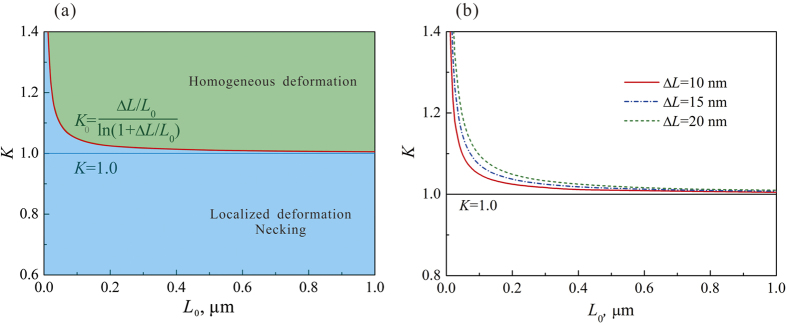
(**a**) Critical normalized strain-hardening rate *K*_0_ for stable and homogeneous plastic deformation under tension plotted as a function of the length of plastic unit *L*_0_ in MGCs. The *K*_0_ line divides the plot area into two sections: under the line *K*_0_, the plastic deformation of MGCs is instable and localized plastic strain or necking occurs; above the line, the plastic deformation is homogeneous elongation occurs. (**b**) Effect of the size of the plastic event (Δ*L*) on the critical normalized strain-hardening rate *K*_0_.

If a stable plastic deformation occurs, the further necking in the plastic unit should be suppressed by the strain-hardening. Therefore, geometrically, the critical normalized strain-hardening rate *K*_0_ is gained as^24^:


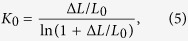


which is the smallest value for stable plastic deformation. In MG materials, the deformation burst Δ*L* can be related to the shear events such as shear transformation zones (STZ) and the minimum thickness of a newly formed shear band^23^,^25^. Therefore, Δ*L* is determined as the plastic event and it is reasonable that Δ*L* is of the order of 10 nm, and the necking length *L*_0_ is changed from 10 nm to several microns^6^,^8^. Given Δ*L* = 10nm, a typical shear event size for metallic glasses^8^,^24^,^26^, we can determine the critical normalized strain-hardening rate against the length of the plastic unit, as shown in Fig. 6a. It shows that the critical normalized strain-hardening rate decreases with increasing length of the plastic unit increasing. In Fig. 6a, the *K*_0_ line based on Eq. 5 separates the plot into two parts. In the lower part of the map (below the *K*_0_ line, i.e. *K* < *K*_0_), the normalized strain-hardening rate is smaller than the critical rate. In this case, the strain-hardening is not large enough to counterbalance the load capability decreasing induced by the area reduction. Therefore, the plastic deformation of the sample is unstable, leading to the necking or localized plastic deformation of the sample. However, in the upper part (above the *K*_0_ line, i.e. *K* > *K*_0_), the normalized strain-hardening rate is sufficiently large to suppress the localized deformation in the plastic unit, resulting in the plastic deformation being effectively transferred to the neighboring undeformed part of the sample. The sequence of this deformation transfer finally results in the homogeneous plastic deformation of the sample. It is interesting that the critical strain-hardening rate for stable plastic deformation can be decreased by decrease the size of the plastic event, as shown in Fig. 6b. When the plastic event decreases from 20 nm to 10 nm, the critical strain-hardening rate is reduced and becomes closer to the line *K* = 1.0. In other words, samples with a larger plastic unit will be more stable than those with small plastic unit under tension.

**Figure 7:**
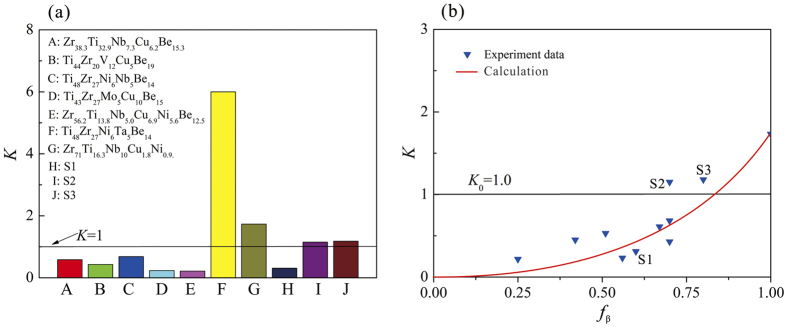
(**a**) Normalized strain-hardening rates of some MGCs with instable and stable plastic deformation under tension. (**b**) Normalized strain-hardening rates of some MGCs with different volume fraction of *β* dendrite.

From Eq. 5, when Δ*L*/*L*_0_ tends to zero, or *L*_0_ tends to be infinite, we get 
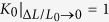
. It also means *θ* = *σ*_0_, which is consistent with the minimum strain-hardening rate for macroscopically stable plastic deformation in traditional materials^19^. Figure 7a shows the average normalized strain-hardening rate of several typical MGCs. It is clear that for the instable *β*-MGCs with necking, their normalized strain-hardening rate is smaller than 1. For example, the normalized strain-hardening rates of two classic *β*-MGCs with chemical compositions of Zr_38.3_Ti_32.9_Nb_7.3_Cu_6.2_Be_15.3_^14^ and Ti_44_Zr_20_V_12_Cu_5_Be_19_^15^ are 0.58 and 0.43, respectively. The normalized strain-hardening rates of the first reported *β*-MGCs of Zr_56.2_Ti_13.8_Nb_5.0_Cu_6.9_Ni_5.6_Be_12.5_^13,20^ and the present *β*-MGC S1 are only 0.22^24^ and 0.31, respectively. However, for the stable *β*-MGCs with homogeneous plastic elongation, the normalized strain-hardening rate is larger than 1. For example, as shown in Fig. 7a, the normalized strain-hardening rate of Ti_48_Zr_27_Ni_6_Ta_5_Be_14_^21^,^22^ is almost 6.0, which is the highest value reported for *β*-MGCs. The normalized strain-hardening rate of the present *β*-MGCs S1 is only 0.31, much smaller than the critical value of 1, which is consistent with its fast plastic instability in the stress-strain relations, as shown in Fig. 2. However, the normalized strain-hardening rates of the present *β*-MGCs S2 and S3 are 1.15 and 1.18. They are slightly larger than the critical value of 1, which is consistent with the mild slope in the early stage of the stress-strain curve of samples S2 and S3. It is noted that the normalized strain-hardening rate of a typical *bcc β* phase with chemical composition of Zr_71_Ti_16.3_Nb_10_Cu_1.8_Ni_0.9_^20^ is only 1.7, which reflects that the reinforcement effect of the *β* phase is too small.

### Effect of volume fraction of β dendrite on plastic deformation of MGCs

As shown in Fig. 2, the plastic stability is strongly dependent on the volume fraction of the *β* dendrites. For a simplistic and ideal condition, provided that the ductile phase yields with the MG matrix synchronically, taking *σ* = *f*_*β*_*σ*_*β*_ + *f*_M_*σ*_M_ (*f*_M_ and *f*_*β*_ are the volume fractions for the MG matrix and the ductile phase; *σ*_M_ and 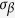 are the active stresses for the MG matrix and the ductile phase, respectively) and 
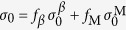
 (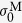 and 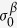 are the yield strengths for the MG matrix and the ductile phase, respectively) into Eq. 4, the normalized strain-hardening rate can be calculated as


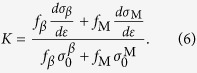


Eq. 6 also can be given as


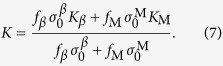


Here, 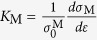 and 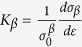 are the normalized strain-hardening rates of the MGC and the ductile phase, respectively. Since *f*_M_ = 1 − *f*_*β*_ and the ratio of the yield strength is 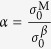, Eq. 7 becomes


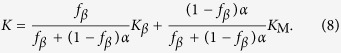


According to Eq. 8, the normalized strain-hardening rate of a *β*-MGC can be calculated based on the experimental data for the two phases. As reported in the literature^24^, if *K* ≥ 1, stable plastic deformation can occur in a *β*-MGC with homogeneous plastic strain, otherwise unstable and localized plastic deformation occurs. Here, by substituting *K*_*β*_ = 1.732 (typical for the *β* phase^24^), *K*_M_ = 0^24^, and*α* = 3.67 in Eq. 8, we obtain *K* as a function of the volume fraction *f*_*β*_ of the *β* dendrites, as shown in Fig. 7b. It is seen that the normalized strain-hardening rate *K* of the *β*-MGC increases monotonically with the volume fraction *f*_*β*_ of the *β* dendrites. When the volume fraction *f*_*β*_ of the *β* dendrites exceeds 0.83, *K* will be larger than the critical value of 1, resulting in the stable and homogeneous plastic deformation of the *β*-MGC under tension. Figure 7b also shows the normalized strain-hardening rate *K* of several reported MGCs with different volume fractions of the ductile phases^14^,^15^,^20^,^21^,^22^,^27^,^28^,^29^,^30^,^31^. It is clear that most *β*-dendrite-reinforced MGCs stay under the line *K*_0_ = 1.0, even when the *β*-dendrite volume fraction of close to 70%.

### Stabilization of plastic deformation in bimetals composed of MGC and pure titanium

Due to the weak strain-hardening capability of *β* dendrites relative to the high strength of the MG matrix, it is very difficult to obtain stable and homogeneous plastic deformation in an MGC. For example, the homogeneous plastic deformation is only 3.46% for sample S3 with the largest *β* volume fraction of more than 80%. If more dendrites are introduced to the MG matrix, the MGC will lose its high strength and the other excellent mechanical properties of the MG matrix. So a practical way is to use MGCs as coatings or multilayers, which can make the most of the excellent properties of the MGs. Here, bimetals composed of pure titanium (MGC/PT) are proposed to improve the stable plastic deformation of an MGC. Similar to Eq. 8, for MGC/PT bimetals, the normalized strain-hardening rate is written as


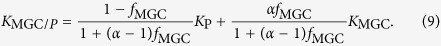


**Figure 8:**
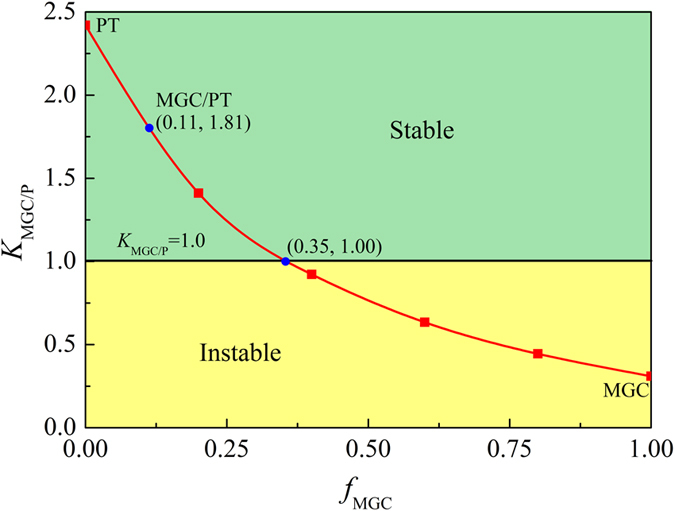
Normalized strain-hardening rate of the bimetal of MGC/PT plotted as a function of the volume fraction of MGC.

Here, *f*_MGC_ is the volume fraction of the *β-*MGC, *K*_P_ the normalized strain-hardening rate of pure titanium, *K*_MGC_ the normalized strain-hardening rate of the *β-*MGC, and *α* the yield strength ratio of the MGC and pure titanium. If *K*_MGC/P_ ≥ 1, stable and homogeneous plastic deformation will occur in the MGC/PT bimetals, otherwise unstable and localized plastic deformation will take place. Here, by substituting *K*_P_ = 2.42 for pure titanium, *K*_MGC_ = 0.31, and *α* = 3.67 into Eq. 9, one can get *K*_MGC/P_ as a function of the thickness fraction *f*_MGC_ for the MGC layer, as illustrated in Fig. 8. It is clear that the normalized strain-hardening rate *K*_MGC/P_ of the MGC/PT bimetals decreases with *f*_MGC_. When *f*_MGC_ is less than 0.35, *K*_MGC/P_ is larger than the critical value of 1, resulting in the MGC/PT bimetals plastically deforming, both stably and homogeneously. For the current MGC/PT bilayer composites with *f*_MGC_ = 0.11, the normalized strain-hardening rate is 1.81, which is well above 1, so its plastic deformation becomes stable and homogeneous, as shown in Fig. 4 and Fig. 8.

Furthermore, the MGC/PT bimetals can be considered as MGCs with a structure similar to other *in-situ* Zr- or Ti-based MGCs with reinforcements of *bcc* dendrites. For the sample with the critical value of the MGC volume fraction (*f*_MGC_ = 0.35) needed for the homogeneous deformation under tension, by counting the PT as reinforcing phase, the actual critical value of the volume fraction of the reinforcing phase is about 86%, which is clearly larger than most of the reported MGCs reinforced by dendrites^14^,^15^,^28^,^29^,^32^. The actual volume fraction of the reinforcing phase of the MGCs with a thickness fraction of 0.11, including the PT substrate, can be as high as 95.6%, which further reflects the limited strain-hardening capability of titanium alloys. Therefore, most *β* dendrite reinforced MGCs are always prone to unstable and localized plastic deformation^14^,^15^,^28^,^29^,^32^.

The deformation mechanism in crystalline materials (dislocation activity) is the major reason why this composite shows such an extended homogeneous plastic strain. The pure titanium yields first, through the activation of a uniformed pattern of dislocation which represents the nucleation site for of multiple shear bands. In this metallic glass composite combined with pure titanium, dislocations can pile up at the crystalline/amorphous interface, which may trigger a shear band in the amorphous layer when the stress concentration at the tip of a pileup reaches the critical yield shear stress^25^. Usually, the pileup is distinguished homogeneously in the order of micrometers along the interface. With the dislocation activity in the pure titanium substrate, the load is transferred to the elastically deforming glassy matrix of the composite. As the pure titanium substrate work hardens, the local stress at the dislocation pileups could be increased to a critical level, which will initiate the shear transformation zones and shear bands in the metallic glass matrix. Thus, a relatively high work hardening rate of the pure titanium stabilizes the plastic flow of the MGC/PT such as to allow synchronous deformation. In order to ensure strain compatibility at the interface, the dislocation governed slip in the PT substrate must be matched by the shear banding in the MGC layer. It is important to know how the dislocations that arrive at the interface in the PT substrate are accommodated in the MGC’s amorphous phase^25^,^33^,^34^. Uniformly spaced dislocation arrays are formed at the interface when glide dislocations on closely spaced planes impinge on the MGC/PT interface. Furthermore, shear bands are nucleated in the glassy matrix of the composite when the shear stress is transferred to the elastically deformed MGC and the local stress reaches the yield shear stress level. At relatively low temperature, these shear transformation zones are nucleated in regions in which there is a critical amount of free volume^23^,^35^,^36^. Thus, a fairly uniform distribution of shear events in the metallic glass composite is induced by the pure titanium layer, which provides accommodation of large homogeneous plastic deformation in the present metallic glass composites.

### Unified plastic deformation modes of MGs and MGCs under tension

**Figure 9:**
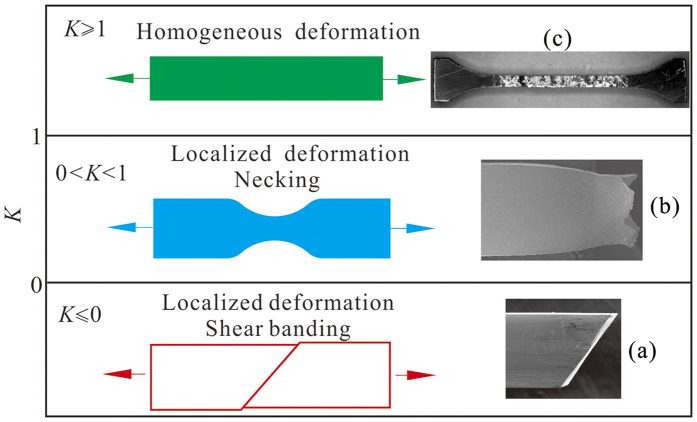
Unified tensile plastic deformation and fracture modes for (**a**) monolithic MGs (*K* ≤ 0), (**b**) MGCs with weak strain-hardening (0 < *K* < 1) and (**c**) MGCs with strong strain-hardening capability (*K* ≥ 1), respectively.

Therefore, the normalized strain-hardening rate is one critical parameter controlling the deformation and fracture modes of MGs and MGCs. For monolithic MGs, their normalized strain-hardening rate is equal or smaller than zero (*K* ≤ 0), or, in other words, there is no strain-hardening, so all the plastic deformation is focused in the narrow shear band, as shown in the lower part of Fig. 9. With the limited addition of reinforcers such as soft *β* dendrites, the normalized strain-hardening rate of the MGCs is larger than zero but smaller than 1 (0 < *K* < 1). In this condition, the strain-hardening rate is not large enough to counterbalance the load capability decrease induced by the area reduction of the MGC samples during tensile elongation. Therefore, there is still no homogeneous plastic deformation, i.e. the plastic strain and strain-hardening can only occur in the localized necking region, as observed in most *β* dendrite reinforced MGCs (see the middle part of Fig. 9). With further increase of the volume fraction of the *β* dendrites, the normalized strain-hardening rate will be equal to or larger than 1, which is large enough to balance the load capability decrease induced by the area reduction during tensile deformation, resulting in homogeneous plastic deformation, as shown in the upper part of Fig. 9.

In summary, the plastic deformation of a series of Ti-based metallic glasses (MGCs) under tension was investigated, and the following conclusions can be drawn. By increasing the volume fraction of the *β* dendrites, the unstable and localized plastic deformation of Ti-based MGCs under tension can be suppressed, and homogeneous plastic deformation under tension can be realized. The strain-hardening rates of Ti-based MGCs were investigated and it was revealed that strain-hardening still occurred in the severely necked Ti-based MGCs. Large homogeneous plastic deformation was reached in the Ti-based MGCs composited with pure titanium. There is a critical value of the normalized strain-hardening rate that determines the plastic stability of Ti-based MGCs: once the normalized strain-hardening rate reaches or surpasses the critical value, the plastic deformation of the Ti-based MGCs is stable, i.e. the necking or strain localization is effectively controlled, resulting in homogeneous plastic elongation in the Ti-based MGCs under tension.

## Methods

### MGCs alloys production

The *β* dendrite reinforced Ti-based MGCs (*β*-MGCs), with chemical compositions Ti_45.4_Zr_26.4_Mo_6_Cu_9.4_Be_12.8_ (labelled sample S1), Ti_50.3_Zr_24.8_Mo_7_Cu_8.3_Be_9.6_ (labelled sample S2), and Ti_55.2_Zr_23.2_Mo_8_Cu_7.2_Be_6.4_ (labelled S3) were prepared by arc melting under an argon atmosphere, and were finally cast in a copper mold. The dimensions of the cast ingots were 5 × 50 mm^2^ (diameter × length). The manufacturing details of bimetals composed of MGC and pure titanium are described in previous reports^37^,^38^.

### Microstructure characterization

The microstructure and chemical composition were examined by a JEM 6490 scanning electronic microscope (SEM) equipped with an energy disperse spectrometer (EDS). The phases of the MGC ingots were characterized by X-ray diffraction (XRD) using a Rigaku diffractometer with Cu *K*_*α*_ radiation. The microstructure was also observed using a Carl Zeiss optical microscope (OM).

### Tensile test

The tensile samples for MGCs with gauge dimensions of 2 × 1 × 1 mm^3^ (length × width × thickness) were prepared by the electric spark method. All laterial surfaces in the tensile sample were ground and finally polished with 0.5 μm diamond paste. The quasistatic tensile tests were conducted using a SANS testing machine at room temperature with a constant initial strain rate of 1 × 10^−4^ s^−1^. Finally, the deformed MGCs samples were investigated by OM and SEM to reveal the deformation and fracture features.

## Additional Information

**How to cite this article:** Wu, F. F. *et al*. Revealing homogeneous plastic deformation in dendrite-reinforced Ti-based metallic glass composites under tension. *Sci. Rep.* **7**, 42598; doi: 10.1038/srep42598 (2017).

**Publisher's note:** Springer Nature remains neutral with regard to jurisdictional claims in published maps and institutional affiliations.
